# Comparison of conventional and non-invasive diagnostic tools for detecting *Plasmodium falciparum* infection in southwestern Cameroon: a cross-sectional study

**DOI:** 10.1186/s40249-021-00859-8

**Published:** 2021-05-22

**Authors:** Tobias O. Apinjoh, Veronica N. Ntasin, Phil Collins C. Tataw, Vincent N. Ntui, Dieudonne L. Njimoh, Fidelis Cho-Ngwa, Eric A. Achidi

**Affiliations:** 1 Department of Biochemistry and Molecular Biology, University of Buea, Buea, Cameroon; 2 Department of Chemical and Biological Engineering, National Higher Polytechnic Institute, The University of Bamenda, Bamenda, Cameroon

**Keywords:** *Plasmodium falciparum*, Microscopy, Nested PCR, Rapid diagnostic test, Blood, Saliva, Urine

## Abstract

**Background:**

Malaria remains a significant health challenge in sub-Saharan Africa, with early diagnosis critical to reducing its morbidity and mortality. Despite the increasing *Plasmodium* spp. diagnostic capabilities, access to testing is limited in some cases by the almost absolute requirement for blood from potentially infected subjects as the only sample source for all conventional methods. A rapid test on non-invasive specimen with comparable performance to microscopy for the screening or diagnosis of all participants is invaluable. This study sought to compare conventional and non-invasive diagnostic tools for detecting *Plasmodium falciparum.*

**Methods:**

This was a cross-sectional study, carried out between March and August 2019 to evaluate and compare the diagnostic performance of a PfHRP2/pLDH-based malaria rapid diagnostic test (mRDT) on patients’ blood, saliva and urine relative to conventional light microscopy and nested PCR at outpatient clinics in the Buea and Tiko health districts of Southwestern Cameroon. The significance of differences in proportions was explored using the Pearson’s *χ*^2^ test whereas differences in group means were assessed using analyses of variance.

**Results:**

A total of 359 individuals of both sexes, aged 1–92 years, were enrolled into the study. Of the 301 individuals tested by light microscopy and mRDTs on blood, saliva and urine, 84 (27.9%), 81 (26.9%), 87 (28.9%) and 107 (35.5%) respectively were positive. However, only 34.3%, 90.5%, 91.4%, 83.9% and 65.4% febrile, light microscopy and mRDT positives on blood, saliva and urine respectively had *P. falciparum* infection as confirmed by PCR. The sensitivity and specificity of presumptive diagnosis, light microscopy and mRDT on blood, saliva and urine were 86.9% and 19.7%, 77.8% and 96.1%, 75.8% and 96.6%, 74.5% and 93.1%, and 70.7% and 81.8%, respectively. The agreement between mRDT on saliva (k = 0.696) and microscopy (k = 0.766) compared to PCR was good.

**Conclusion:**

The study highlighted the low performance of presumptive diagnosis, reinforcing the need for parasitological tests prior to antimalarial therapy. The higher PfHRP2/pLDH mRDT parasite detection rates and sensitivity in saliva compared to urine suggests that the former is a practical adjunct to or alternative worth optimising for the routine diagnosis of malaria.

**Graphic Abstract:**

Flow chart for diagnosis of *P. falciparum* infection by light microscopy, rapid diagnostic tests and nested PCR.
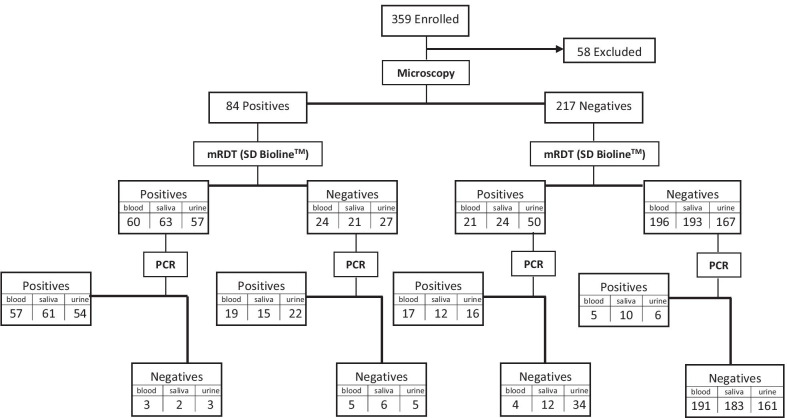

## Background

Malaria remains a significant health problem especially in sub-Saharan Africa despite a steady decrease in cases and deaths over the last decade [1]. The disease burden is exacerbated in rural developing country settings with few qualified healthcare providers, ill-equipped health facilities and often limited electricity supply [2, 3]. In Cameroon, almost 4 million suspected malaria cases still report to health facilities annually [4] despite the sustained scale-up of proven and highly effective malaria control interventions.

Early diagnosis and treatment remains critical to preventing death and reducing the disease and its transmission [1, 5]. Although empiric/syndromic diagnosis is one widely used method based on clinical algorithms [6], presumptive diagnosis, the deep-rooted practice of treating all fevers as malaria, overdiagnoses the disease [7], leading to substantial economic losses [8] and the possibility of selecting resistant parasite strains following the deployment of artemisinin combination therapy (ACT) [9]. Current malaria management guidelines recommend prompt parasitological confirmation of all suspected malaria patients by microscopy and/or rapid diagnostic test (RDT) prior to antimalarial treatment [5]. Although laboratory diagnosis of malaria traditionally involves the microscopic examination of Giemsa-stained blood smears and remains the gold standard tool [10, 11], its limitations, including the need for skilled technicians and electricity supply [6] necessitated the development of RDTs [10].

Rapid diagnostic tests are vastly sensitive and specific compared with microscopy but also compare favourably with the polymerase chain reaction (PCR) [12], the most sensitive molecular technique for detecting parasites [13]. RDTs are limited mainly by their inability to detect infections at low parasitaemia. In addition, false positive or negative results may be reported due to the persistence of target antigen even after successful treatment or deletions in the PfHRP2 gene respectively [10, 14]. Nevertheless, they are affordable, easy-to-perform, fast, reliable and effective diagnostic point-of-care tools for malaria case management especially in endemic rural settings and areas with limited laboratory facilities [2].

Despite increasing *Plasmodium* spp. diagnostic capabilities, access to testing is challenged, particularly in community and private healthcare settings, by the almost absolute requirement for blood from potentially infected subjects as the sample source for all methods [6, 13]. Blood collection entails invasive procedures with associated side effects such as bruising or pain and haematoma but sometimes serious complications [15]. In addition, blood taboos, concerns about needle-stick injuries and disease transmission [16] and lack of compliance from vulnerable individuals when repeated sampling is needed [17] continue to limit malaria diagnosis and bar participant enrolment in biomedical research [18]. A non-invasive malaria rapid diagnostic tool that exploits other body fluids will be invaluable for healthcare delivery especially in peripheral institutions.

Saliva and urine are two common alternatives to blood for the diagnosis of infectious diseases that are readily available and easy to collect. Saliva has been effectively used to detect the 2019 novel coronavirus, SARS-CoV-2 [19] and the human immunodeficiency virus [20] as well as to assess immunity to measles, mumps, rubella and hepatitis [21, 22]. The usefulness of both saliva and urine in the diagnosis of malaria in endemic settings has been documented using sensitive PCR-based approaches to detect *P. falciparum* infection [23, 24], parasite DNA [25] and *P. falciparum* histidine-rich protein 2 (*Pf*HRP2) antigen [26, 27] in samples from human malaria patients. There are however, limited reports evaluating the performance of RDTs in the non-invasive diagnosis of malaria [28, 29]. Considering the variability of malaria diagnostic tests in different transmission settings and the need for a rapid test on non-invasive specimen with comparable performance to microscopy for the screening or diagnosis of all participants, this study evaluated the diagnostic performance of a commercially available mRDT on blood, saliva and urine from the same patients at outpatient clinics in the Buea and Tiko health districts of Southwestern Cameroon.

## Methodology

### Study area

This study was carried out in the Buea (4° 10′ 0″ N 9° 14′ 0″ E) and Tiko (4° 4′ 30″ N 9° 21′ 36″ E) health districts, located at the foot of mount Cameroon in the Southwestern Region of Cameroon. The area has an equatorial climate characterized by daily temperatures ranging between 20 and 33 °C, average annual rainfall of 2625 mm, relatively high humidity and precipitation and two major seasons; a long rainy season (March–November) and a short dry season (November–March) [30]. *Plasmodium* spp. transmission is perennial in this area, with *Anopheles gambiae* being the dominant vector species and *Plasmodium falciparum* accounting for most malaria infections [31].

### Study design, population and sampling

This cross-sectional study was carried out in the Regional Hospital, Buea and the Baptist Hospital, Mutengene from March to August 2019. A sample size of 293 participants was estimated using the general formula for comparing two independent proportions of alternative diagnostic tests, assuming 95% confidence interval and 80% power to detect a difference of 10% from a sensitivity or specificity of at least 70% as described [32]. Individuals of both sexes and all ages, resident at different altitudinal zones as described previously [30] and reporting for malaria test in the outpatient departments were eligible for enrolment. Individuals who did not consent or were unable to provide all samples were excluded. A structured questionnaire was used to document demographic and clinical data, followed by venous blood (about 2 ml), saliva (about 3 ml) and urine (about 20 ml) collection from the same patient into labelled ethylene diamine tetraacetate and sterile dry tubes respectively. All samples were stored between 2 and 8 °C on ice blocks until analysis. The malaria parasite status in blood, saliva and urine was immediately checked using mRDT, blood smears prepared on grease-free slides as described [33] and about 50 µl of blood spotted on filter paper for *P. falciparum* detection using PCR.

### Laboratory analysis

#### Haemoglobin measurement

Haemoglobin (Hb) concentrations were determined using a hemoglobinometer (Hangzhou Sejoy Electronics, Hangzhou, China) and anemia was defined as Hb < 11.0 g/dL [34].

#### Malaria rapid diagnostic testing

The presence of *Plasmodium* spp. in blood, saliva and urine was assessed using the PfHRP2/pLDH malaria rapid diagnostic kit (SD Bioline, Alere, South Korea) and according to the manufacturer’s instructions. Briefly, 5 µl of sample from each was placed in the sample window of the RDT cassette and three drops of diluent added. The results were then read after 15 min, with the presence of two (or three), one or no distinct line indicative of a positive, negative or invalid result respectively.

#### Light microscopy

Thin and thick blood smears were prepared and allowed to air dry. The thin films were fixed with methanol and both smears stained with 10% Giemsa for 15 min as described [33]. Subsequently, the stained slides were then air-dried and viewed under the 100 × oil immersion objective of a binocular Olympus microscope. Each slide was examined by two independent microscopists and was considered negative if no parasite was seen after counting 500 white blood cells. With each positive slide, the parasite densities were calculated by counting the parasites against a minimum of 200 leucocytes and assuming an average leucocyte count of 8000 per µl of blood [11].

#### DNA extraction and PCR

DNA was extracted from dried blood on filter paper using the Chelex method as previously described [35], with some modifications. Each filter paper punch in 1 ml of 0.5% saponin was incubated overnight at 4 °C to lyse red blood cells. The punch was then washed in 1 ml of 1% phosphate buffer saline, incubated at 4 °C for 30 min and then transferred into a new tube containing 50 µl of 20% Chelex and 150 µl distilled water on a thermomixer. The tube was then heated at 99 °C for 10 min to elute the DNA, vortexed for about 2 min and then centrifuged at 11 000×*g* for another 2 min. The supernatant (DNA extract) was then transferred into new Eppendorf tube and kept at 4 °C for use within hours or at − 20 °C for long term storage.

The *P. falciparum* 18S rRNA gene was then amplified by nested PCR as described previously [36] using specific predesigned primers (Table 1).

**Table 1:** PCR primers for *Plasmodium falciparum* detection

Gene	Primers	Sequence	Band size (bp)
Primary PCR			
Mitochondrial *coxI* gene	rPLU5	5′-GACCTGCATGAAAGATG-3′	595
	rPLU6	5′-GTATCGCTTTAATAGGCG-3′	
Nested PCR			
18S rRNA	fal1	5′-GGAATGTTATTGCTAACAC-3′	205
	fal2	5′-AATGAAGAGCTGTGTATC-3′	

All PCR amplifications were carried out on a thermal cycler (BioRad, CA, USA), in a total volume of 15 µl, consisting of 7.5 µl of 2 × *Taq* master mix, 0.5 µl each of forward and reverse primers and 4.5 µl of nuclease free water and 2 µl of extracted genomic DNA (primary PCR) or 5.5 µl of nuclease free water and 1 µl of primary PCR product (nested reaction). The cycling conditions of both reactions were the same, except that the annealing temperature and the number of cycles were increased from 55 to 61 °C and 25 to 30 during the nested reaction. The nested PCR products were visualized on a UV transilluminator and a molecular imager relative to a 100 bp molecular weight marker, following ethidium bromide stained 2.5% agarose gel electrophoresis in 0.5 × TAE buffer using a power pack (Biorad, CA, USA) at 100 V for 25 min. A sample was considered positive if a 205 bp band was detected. *P*. *falciparum* 3D7 DNA and distilled water served as positive and negative controls, respectively in every set of reactions.

### Data analysis

Data obtained was analyzed using IBM SPSS Statistics 20 (SPSS Inc, Chicago USA). Patients without results for any diagnostic test or with indeterminate PCR results were excluded from the analysis. Levels of parasitaemia were log-transformed before analysis. The significance of differences in proportions of qualitative variables was explored using the Pearson’s *χ*^2^ test whereas the differences in group means were assessed using analyses of variance. The performance of each diagnostic test was assessed by estimating the sensitivity, specificity, positive and negative predictive values using the following formula and their respective 95% confidence intervals as well as the Chi-square goodness of fit of each technique. Kappa test was also used to determine the degree to which RDT compares to microscopy and PCR. A probability (*P*) value < 0.05 was considered as statistically significant at a 95% confidence interval.

Sensitivity = true positive/(true positive + false negative) × 100%

Specificity = true negative/(true negative + false positive) × 100%

Positive predictive value (PPV) = true positive/(true positive + false positive) × 100%

Negative predictive value (NPV) = true negative/(true negative + false negative) × 100% [28].

## Results

A total of 359 individuals of both sexes, aged 1–92 years, were enrolled in this study. The characteristics of the population are summarized in Table 2. Most participants were adults (81.7%), females (71.8%), febrile (82.4%), non anaemic (63.8%), non bednet users (64.1%), resident at high altitude (47.8%) and from the Semi-Bantu ethnic group (58.1%). Eighty-four and 81 individuals were malaria parasite positive based on routine light microscopy and mRDT on blood respectively, while only 60 participants were positive by both methods. Fifty-eight participants were excluded from further analysis because results of one or more diagnostic tests could not be obtained.

**Table 2:** Demographic, clinical and blood parasitological characteristics of the study population

Variable	Overall (*n* = 301)	Children (*n* = 55)	Adolescents (*n* = 32)	Adults (*n* = 213)	Level of significance
*Patient characteristics, Mean ± SD [range]*					
Age (years)	29.8 ± 17.1 [1–92]	7.7 ± 4.6 [1–14]	18.3 ± 1.8 [15–20]	37.3 ± 14.3 [21–92]	F = 142.83; *P* < 0.001
Weight (kg)	61.2 ± 20.0 [10–124]	28.8 ± 16.0 [10–72]	53.9 ± 12.5 [18.1–77.0]	71.0 ± 11.3 [35.0–124]	F = 255.88; *P* < 0.001
Temperature (°C)	37.7 ± 0.9 [35.0–41.0]	38.1 ± 1.0 [35.0–39.8]	37.8 ± 1.1 [35.6–40.5]	37.5 ± 0.8 [35.4–41.0]	F = 9.14; *P* < 0.001
Haemoglobin level (g/dL)	11.5 ± 1.4 [8.2–16.9]	11.2 ± 1.3 [9.2–14.0]	11.3 ± 1.5 [9.0–16.9]	11.6 ± 1.4 [8.2–15.1]	F = 1.76; *P* = 0.174
^¥^GMPD (parasites/µl blood)	849 [105–7200]	695 [200–3500]	680 [105–4500]	942 [200–7200]	F = 0.758; *P* = 0.472
*Patient characteristics,* *n* (%)					
Proportion of females (%)	216 (71.8)	31 (56.4)	23 (71.9)	162 (76.1)	*χ*^2^ = 8.41; *P* = 0.015
Fever; temperature ≥ 37.5 ℃	166 (55.1)	41 (74.5)	18 (56.2)	106 (49.8)	*χ*^2^ = 10.87; *P* = 0.004
History of fever	212 (70.4)	50 (90.9)	23 (79.1)	138 (64.8)	*χ*^2^ = 14.33; *P* = 0.001
Febrile (fever and/or history of fever)	248 (82.4)	54 (98.2)	25 (78.1)	168 (78.9)	*χ*^2^ = 11.64; *P* = 0.003
Anaemia (%)	109 (36.2)	23 (41.8)	85 (34.7)	85 (34.7)	*χ*^2^ = 1.18; *P* = 0.320
Bednet usage (%)	108 (35.9)	22 (40.0)	85 (34.7)	85 (34.7)	*χ*^2^ = 0.68; *P* = 0.458
Positive by microscopy	84 (27.9)	17 (30.9)	11 (34.4)	56 (26.3)	*χ*^2^ = 1.19; *P* = 0.553
Positive by mRDT	81 (26.9)	12 (21.8)	10 (31.2)	59 (27.1)	*χ*^2^ = 1.10; *P* = 0.578
Positive microscopy & positive mRDT	60 (19.9)	9 (16.4)	9 (28.1)	42 (19.7)	*χ*^2^ = 6.27; *P* = 0.394
Positive microscopy & negative mRDT	24 (8.0)	8 (14.5)	2 (6.2)	14 (6.6)	*χ*^2^ = 6.27; *P* = 0.394
Negative microscopy & positive mRDT	21 (7.0)	3 (5.5)	1 (3.1)	17 (8.0)	*χ*^2^ = 6.27; *P* = 0.394

^¥^*GMPD* Geometric mean parasite density; anaemia = Hb < 11.0 g/dL; *mRDT* Malaria Rapid Diagnostic Test; Children = 0–14 years; Adolescents = 15–20 years; Adults ≥ 21 years; The Age of one participant was not known; The significance of differences in proportions were explored using the Pearson’s *χ*^2^ test while differences in group means were assessed using analyses of variance

### Diagnostic test results

**Fig. 1:**
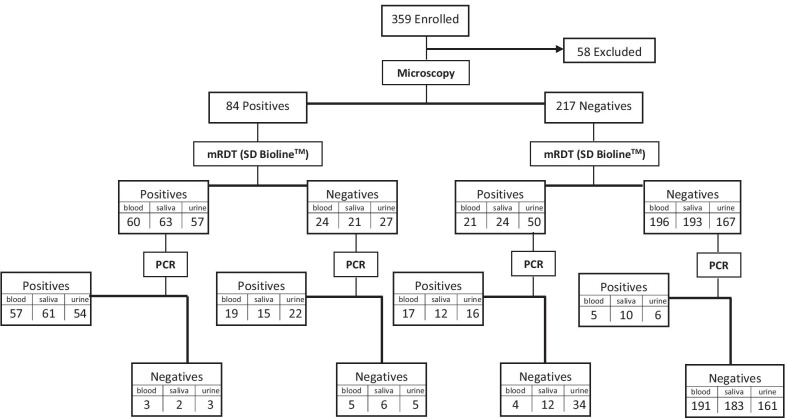
Flow chart for diagnosis of *Plasmodium falciparum* infection by light microscopy, rapid diagnostic tests and nested PCR

**Fig. 2:**
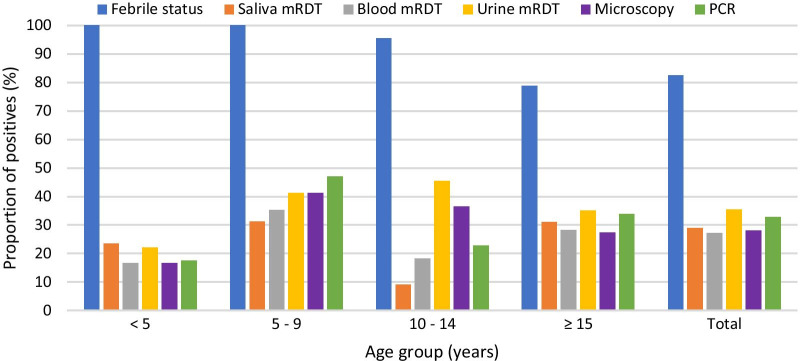
Distribution of *Plasmodium falciparum* infection by age group as assessed by different diagnostic tools. *mRDT* malaria rapid diagnostic test; *PCR* polymerase chain reaction

Of the 301 participants tested for *P. falciparum* infection, 98 (32.6%) were positive by PCR while 84 (27.9%), 81 (26.9%), 87 (28.9%) and 107 (35.5%) cases were detected by light microscopy and mRDTs on blood, saliva and urine respectively. Majority (248, 82.4%) of the participants had axillary temperature ≥ 37.5 °C and/or history of fever at enrollment, with 13 (24.5%) and only 85 (34.3%) of the afebrile and febrile individuals respectively having a positive test for *P. falciparum* by PCR. The detailed result of the trial profile of the five diagnostic methods is shown in Fig. 1. Of the 84 microscopy positive samples, 60, 63 and 57 were positive following mRDT on blood, saliva and urine; and 3, 2 and 3 respectively confirmed negative for *P. falciparum* by PCR. Conversely, of the 217 microscopy negative participants, 196, 193 and 167 respectively were negative following mRDT on blood, saliva and urine; and 5, 10 and 6 shown to be *P. falciparum* infected after PCR (Fig. 1). The trends in fever and malaria parasite positivity by all five diagnostic methods were similar amongst the different age groups (Fig. 2), with the odds of being diagnosed positive highest in the 5–9 and > 15 years age group and lowest in children below 5 years of age. There was an association (*χ*^2^ = 11.82, *P* = 0.008) between febrile status and age group, with all children below 9 years of age, having a febrile illness at enrolment. The proportion of malaria parasite infected participants as detected by microscopy (*P* = 0.332), blood mRDT (*P* = 0.459), saliva mRDT (*P* = 0.171), urine mRDT (*P* = 0.458) and PCR (*P* = 0.215) was independent of age.

### Performance of malaria diagnostic methods compared with PCR

PCR is the most sensitive diagnostic technique and therefore the reference standard in this study. As such, participants found to be positive by any other diagnostic method, but not by PCR, were considered “false” positive. PCR confirmed 34.3% (85/248) febrile and 90.5% (76/84) microscopy positive individuals as well as 91.4% (74/81), 83.9% (73/87) and 65.4% (70/107) of mRDT positives on blood, saliva and urine respectively as *P. falciparum*-infected. Thus, among the samples thought to be infected with the malaria parasite on the basis of the participant being febrile or diagnosed positive by light microscopy or via mRDT on blood, saliva or urine, 163 (65.7%), 8 (9.5%), 7 (8.6%), 14 (16.1%) and 37 (34.6%), respectively were confirmed to be *P. falciparum* negative by PCR. Furthermore, 24.5% (13/53) of the afebrile as well as 10.1% (22/217), 10.9% (24/220), 11.7% (25/214) and 14.4% (28/194) malaria parasite uninfected participants by microscopy, and mRDT negative on blood, saliva and urine respectively had *P. falciparum* infection*.* The sensitivity and specificity of all five test methods, with their corresponding positive and negative predictive values are shown in Table 3.

**Table 3:** Diagnostic statistics for malaria tests compared to the reference method and gold standard

Test	Sensitivity % (95% *CI*)	Specificity % (95% *CI*)	PPV % (95% *CI*)	NPV % (95% *CI*)	Kappa
*PCR*					
Febrile status	86.9 (80.2–93.5)	19.7 (14.2–25.2)	34.5 (28.6–40.4)	75.5 (63.9–87.1)	0.020
Microscopy	77.8 (69.6–86.0)	96.1 (93.3–98.7)	90.6 (84.4–96.8)	89.9 (85.8–93.9)	0.766
mRDT (blood)	75.8 (67.3–84.2)	96.6 (94.0–99.1)	91.5 (85.4–97.5)	89.1 (85.0–93.2)	0.175
mRDT (saliva)	74.5 (65.9–83.1)	93.1 (89.6–96.6)	83.9 (76.2–91.6)	88.3 (84.0–92.6)	0.696
mRDT (urine)	70.7 (61.7–79.7)	81.8 (76.5–87.1)	65.4 (56.4–74.4)	85.1 (80.1–90.1)	0.514
*Light microscopy*					
Febrile status	88.2 (81.4–95.1)	19.7 (14.4–25.0)	30.0 (24.3–35.7)	81.1 (70.6–91.7)	0.021
mRDT (blood)	71.8 (62.2–81.3)	90.4 (86.5–94.3)	74.4 (64.9–83.8)	89.1 (85.0–93.2)	0.136
mRDT (saliva)	75.0 (65.7–84.3)	88.9 (84.8–93.1)	72.4 (63.0–81.8)	90.2 (86.2–94.2)	0.632
mRDT (urine)	67.1 (57.1–77.1)	77.1 (71.5–82.6)	53.3 (43.8–62.7)	85.7 (80.8–90.6)	0.409

*CI* confidence interval; *mRDT* malaria rapid diagnostic test; *NPV* negative predictive value; *PCR* polymerase chain reaction; *PPV* positive predictive value

Cohen’s kappa coefficient was used to assess the agreement between tests and the most sensitive method, PCR (Table 3). As such, febrile status (k = 0.020) and blood mRDT (k = 0.175) compared poorly with PCR while there was a moderate agreement with urine mRDT (k = 0.514) and good agreement with saliva mRDT (k = 0.696) and microscopy (k = 0.766) when compared to PCR. The level of agreement was similar when the malaria diagnostic tests were compared with the gold standard microscopy; poor agreement was obtained with febrile status (k = 0.021) and blood mRDT (k = 0.136) while moderate and good agreement was obtained with urine mRDT (k = 0.409) and saliva mRDT (k = 0.632) respectively (Table 3). The agreement between both (k = 0.153 each) saliva and urine mRDT as well as febrile status (k = 0.054) was poor when compared to blood mRDT. There was a significant difference (*P* < 0.001 each) in the performance of mRDT on both saliva and urine when compared to blood mRDT.

### Sensitivity of malaria diagnostic methods at different levels of peripheral *P. falciparum* parasitemia

Variation of the sensitivity (based on febrile status and mRDT diagnosis) with *P. falciparum* parasitaemia recorded by light microscopy is shown (Table 4). The highest sensitivities were recorded at parasite densities of more than 500 parasites per microliter; with 75.6%, 77.8%, 86.7% and 91.1% sensitivity for mRDT on urine, blood and saliva as well as diagnosis based on fever and/or history of fever at enrolment respectively. The performance of all diagnostic tools dropped at lower parasite densities.

**Table 4:** Sensitivity of febrile status and mRDT compared to PCR at different levels of peripheral *Plasmodium falciparum* parasitemia

Parasite threshold	*n*	Sensitivity, % (95% *CI*)			
		Febrile individuals	mRDT (blood)	mRDT (saliva)	mRDT (urine)
NPS	217	86.4 (72.0–100)	77.3 (59.8–94.8)	54.5 (33.7–75.3)	72.7 (54.1–91.3)
≤ 500	37	80.6 (66.7–94.6)	71.0 (55.0–86.9)	71.0 (55.0–86.9)	64.5 (47.7–81.4)
> 500	47	91.1 (82.8–99.4)	77.8 (65.6–89.9)	86.7 (76.7–96.6)	75.6 (63.0–88.1)

*CI* confidence interval; *mRDT *Malaria Rapid Diagnostic Test; *NPS *no parasite seen

## Discussion

The requirement of blood for almost all malaria diagnostic tests limits access mainly due to concerns about disease transmission, blood taboos, needle-sticks injuries [16] and compliance issues in severely anaemic participants or vulnerable groups when repeat sampling is needed [17]. A non-invasive malaria rapid diagnostic tool with comparable performance to microscopy for the screening or diagnosis of all participants, that exploits other readily available body fluids will, therefore, be invaluable for healthcare delivery as well as increase patronage in biomedical research [18]. The study evaluated the performance of a commercially available rapid antigen detection test, adopted by the Cameroon national malaria control program, for the diagnosis of malaria in saliva and urine and compared this to conventional methods in use at outpatient clinics in the Buea and Tiko health districts of Southwestern Cameroon.

The use of a commercial blood PfHRP2/pLDH detection test to effectively identify malaria parasite-infected individuals in saliva and urine confirms limited reports of the utility of mRDT as non-invasive, non-microscopic yet rapid tools for the detection of the parasite [28, 29]. The release of malaria parasite antigen into the saliva is thought to be associated with fever that leads to vasodilation of vessels supplying the buccal cavity and gingivitis that release blood into the mouth [28]. The small size of PfHRP2/pLDH suggests that they may be freely excreted in urine [37, 38], since the kidney has been implicated in falciparum malaria [39], leading to the release of specific malaria proteins [28] and even larger molecules such as albumin [40].

Although a higher sensitivity has been recorded in urine samples from India [41], detection rates and sensitivities for mRDT on saliva and urine recorded in this study were higher than previously reported across sub-Saharan Africa [28, 29]. This may be due to higher antigen levels in the samples and/or their minimal degradation since all specimens were analysed almost immediately after collection [24] and not stored [42] or spun [27] to potentially reduce the antigen concentration or activity prior to analysis. This fresh whole saliva/urine approach has been shown previously [29] to be a more sensitive and practical alternative for routine diagnosis of malaria in resource-limited endemic settings. Furthermore, saliva was more sensitive and specific compared to urine overall, with sensitivity at parasitaemia greater than 500 parasites/µl comparable to that obtained in Nigeria using diagnostic kits specifically designed to detect malaria antigen in urine [43, 44]. Saliva is, therefore, a better non-invasive alternative fluid for the rapid diagnosis of malaria [28] worthy of further investigation as an adjunct to microscopy, since its sensitivity is still below optimum. Such sensitive tools are relevant in endemic settings where potentially life-threatening febrile illnesses abound while high specificity will minimise unnecessary antimalarial use. Nevertheless, the sample source still warrants further investigation prior to its use in clinical practice, as mRDT was unable to detect parasite antigen in nine saliva samples in spite of matching whole-blood mRDT, microscopy and PCR positive samples from the same participants. It remains unclear why such disparities exist, although differences in antigen concentration in saliva and whole blood have been incriminated [29].

This study also assessed the performance of different methods in detecting malaria parasite infection among febrile participants, with PCR outperforming blood smear microscopy and HRP2-based malaria RDT as reported previously [45, 46], detecting five samples that were negative by all other methods. With 10.1% of sub-microscopic infections identified in this study, the relevance of PCR and other more sensitive molecular techniques in complementing the clinical diagnosis of malaria and addressing the misdiagnoses of low-density infections and mixed parasite infections [47] cannot be overemphasized. This threat to clinical diagnosis and malaria control in the area and beyond will only increase as transmission declines [48] and elimination nears. However, some five microscopy positive samples were negative by nested PCR, possibly due to mutation of the sequence recognised by predesigned primers that renders target gene amplification impossible [13] although this may also reflect limited microscopy competency [49]. Reports of genome-wide gene deletions in *P. falciparum* [50] suggests that the parasite may delete the target as a strategy to enhance its survival and spread.

Overall, the proportion of confirmed malaria parasite infected individuals was low (32.6%) in spite of the high proportion (82.4%) of participants with axillary temperature ≥ 37.5 °C and/or history of fever at enrollment. This finding is consistent with previous reports [51] and is suggestive of a reduced malaria burden in the area, accruing to the population’s adherence to national malaria control and prevention strategies [52].

Although the sensitivity of presumptive diagnosis was quite high, the extremely low specificity recorded in this study vastly overdiagnoses the disease [11, 53]. This will lead to substantial economic losses [8] and the possibility of selecting resistant parasite strains, if ACT or other recommended antimalarials are deplored to treat these fevers [9] since there is a very high chance of treating people without malaria [7]. This challenge reinforces the need to adhere to the current malaria management guidelines that strongly recommend prompt parasitological confirmation of all suspected malaria patients by microscopy and/or RDT prior to antimalarial treatment. Treatment on the basis of clinical suspicion should only be considered if such diagnosis is inaccessible and the probability exist that the illness is malaria [5].

Although light microscopy agreed pretty well with PCR as reported previously [47], a 9.4% false negative rate was recorded with this gold standard diagnostic tool in this study, highlighting the need for a backup check in the routine diagnosis of malaria in all healthcare settings. Although higher values have been recorded [12, 45], the sensitivity and specificity of the tool compared to PCR mirrored previous studies [44], and was better than previous reports [47, 51]. Such discrepancies may accrue to the subjective nature of the test that depends on the training and experience of the microscopist but also the quality and storage of supplies [49]. There is therefore the need for the national malaria control and elimination programs and management of healthcare facilities to provide comprehensive and continued training and retraining to laboratory personnel and ensure gold standard supply service to reduce the morbidity, mistreatment, potential mortality and expenditure from malaria [2, 49]. In spite of these limitations, and the perennial lack of constant electricity supply and equipment especially in rural endemic settings, microscopy remains the gold standard in clinical practice.

RDTs in combination with microscopy would improve malaria diagnosis**.** The use of HRP2-based mRDT (SD Bioline) in this study was informed by the recommendation of HRP2-based assays in areas where *P. falciparum* is predominant [54] and reports of its greater sensitivity relative to other rapid diagnostic tests in the area [12] and elsewhere in the country [55]. However, the mRDT apparently over-diagnosed malaria parasite infection in four blood/urine and two saliva samples. Such “false positive” mRDT results are consistent with previous reports of the detection of HRP2 antigenemia before microscopic and PCR-positive conversion in falciparum malaria [56] perhaps due to parasite sequestration in deep capillaries of the bone marrow, liver or spleen or rapid clearance by the immune system early in infection. Furthermore, mRDT detected antigen (in all three sample sources) from twelve PCR confirmed malaria parasite infected participants that were diagnosed negative by microscopy. This may be due to immune activation, submicroscopic parasitaemia or suggestive of recently treated malaria infection [45], since four of these participants reportedly took drugs prior to enrolment. These treated participants remain mRDT positive since the HRP2 antigen remains in circulation post-treatment for about a month whereas, the antimalarial drugs would have effectively disintegrated the parasites, which will be undetectable by blood smear microscopy [57]. Patients with positive mRDT results discordant with microscopy and PCR must therefore be carefully evaluated, frequently followed-up and tested with multiple methods for accurate diagnosis [56].

However, the mRDT could not detect the target antigen in eight matching saliva/blood samples with microscopy and PCR positive results. These false negatives may be due to the low parasitaemia (310–3500 parasites/µl blood) recorded in this study, consistent with low multiplicity of infection and/or malaria parasitaemia in the area [30, 34]. They may also be due to low target antigen expression typical of infections from low transmission settings such as the high altitude areas where majority of the participants were enrolled, gene deletions that prevent antigen expression [14], sequence variation or polymorphisms in target antigen that affect the protein binding to monoclonal antibodies on the test kit and prozone effects or other factors [58, 59]. WHO recommends studies to determine the prevalence of genetic mutations/HRP2 deletions in field isolates since these are likely not the main reason behind false negative mRDT results [60].

When compared with PCR, the sensitivity of mRDT though consistent with previous reports in areas of low transmission [45, 51] was below the WHO recommended threshold of 90%. The accuracy of the RDT in detecting malaria parasites may have been influenced by the concentration of target antigen in the sample, dynamics of antigen–antibody flow through the test strip, availability of specific epitopes to bind antibodies in the test [42], storage and distribution, target antigen stability, circulating parasite species [54], climate and level of transmission [61]. Although mRDT doesn’t appear to be an ideal malaria diagnostic tool in this context, it is a readily available and cost-effective alternative to microscopy for rapid and better management of malaria [62]. As such, health personnel may need to investigate positive mRDT tests, since febrile episodes of individuals, especially children, seeking healthcare from rural resource-limited settings often accrue to multiple infections and strict adherence to malaria rapid test results might lead to a neglect of other underlying diseases [63].

The study had a number of limitations. Firstly, the mRDT and nested PCR diagnostic tools could only detect *P. falciparum* but not other species such as *P. malariae* and *P. ovale* that have been reported in the area [12, 31], possibly underestimating the actual proportion of infected individuals. Secondly, prior use of antimalarial by some participants may have contributed to the false-positive mRDT results recorded in this study.

## Conclusions

This study highlighted the low performance of presumptive diagnosis and the need for parasitological tests prior to antimalarial therapy. A commercial blood PfHRP2/pLDH detection test was effectively used to identify malaria parasite-infected individuals using saliva and urine samples. Although still below optimum as recommended by WHO, saliva had higher detection rates and sensitivity compared to urine, making it a practical adjunct to or alternative worth optimising for the routine diagnosis of malaria. Overall, the proportion of confirmed malaria parasite infected cases was low, suggestive of a reduced malaria burden in the area perhaps due to the population’s adherence to national malaria control and prevention strategies.

## Data Availability

All data generated or analysed during this study are included in this published article.

## Abbreviations

ACT: Artemisinin combination therapy
*CI*: Confidence interval
GMPD: Geometric mean parasite density
mRDT: Malaria rapid diagnostic test
NPS: No parasite seen
NPV: Negative predictive value
PCR: Polymerase chain reaction
PfHRP2: *Plasmodium falciparum* histidine rich protein 2
pLDH: *Plasmodium* lactate dehydrogenase
PPV: Positive predictive value
WHO: World Health Organisation
